# Digital in-line holographic microscopy for label-free identification and tracking of biological cells

**DOI:** 10.1186/s40779-024-00541-8

**Published:** 2024-06-13

**Authors:** Jihwan Kim, Sang Joon Lee

**Affiliations:** Department of Mechanical Engineering, Pohang University of Science and Technology, Pohang, Gyeongbuk 37673 Republic of Korea

**Keywords:** Digital in-line holographic microscopy (DIHM), Cell identification, Cell tracking, Artificial intelligence

## Abstract

Digital in-line holographic microscopy (DIHM) is a non-invasive, real-time, label-free technique that captures three-dimensional (3D) positional, orientational, and morphological information from digital holographic images of living biological cells. Unlike conventional microscopies, the DIHM technique enables precise measurements of dynamic behaviors exhibited by living cells within a 3D volume. This review outlines the fundamental principles and comprehensive digital image processing procedures employed in DIHM-based cell tracking methods. In addition, recent applications of DIHM technique for label-free identification and digital tracking of various motile biological cells, including human blood cells, spermatozoa, diseased cells, and unicellular microorganisms, are thoroughly examined. Leveraging artificial intelligence has significantly enhanced both the speed and accuracy of digital image processing for cell tracking and identification. The quantitative data on cell morphology and dynamics captured by DIHM can effectively elucidate the underlying mechanisms governing various microbial behaviors and contribute to the accumulation of diagnostic databases and the development of clinical treatments.

## Background

Recent advancements in microscopy technology have facilitated quantitative analysis of cell dynamics in the fields of cell biology and biomedical research [1–5]. The utilization of miniaturized total analytic systems and lab-on-a-chip technologies has increasingly enabled digital imaging techniques to analyze the dynamic behaviors of living cells under various microfluidic conditions [6–10]. For example, optical microscopy has been employed to analyze membrane deformation and rotational motion of erythrocytes flowing in microchannels [11–16]. Confocal microscopy was utilized to reconstruct the three-dimensional (3D) morphological structures of various biological samples from their high-resolution stacks of images obtained by point-by-point scanning using a focused illuminating beam [17–20]. This advanced microscopy technique allowed for the analysis of cell dynamics under different microenvironments. Defocusing microscopy was used to estimate the 3D surface characteristics of cells under various experimental conditions [21–23]. However, these microscopy techniques have technical limitations in observing cell dynamics within a wide 3D volume due to their limited depth of field (DOF).

Digital in-line holographic microscopy (DIHM) is a 3D imaging technique that efficiently captures both the 3D positional and morphological information of test samples over time [24–32]. Unlike conventional microscopy techniques, which only provide two-dimensional (2D) images on the focal plane of an objective lens, DIHM records consecutive holographic interference signals of test samples that contain 3D volumetric information using a digital image recording device, such as a charge-coupled device camera or a complementary metal-oxide-semiconductor camera. By employing various numerical backpropagation methods, holographic images at different distances from the sensor plane of the DIHM system are reconstructed. Autofocusing algorithms are subsequently applied to determine the 3D positions of test samples and obtain the corresponding in-focus reconstructed images. Four-dimensional (4D; 3D spaces + 1D time) spatio-temporal trajectories of test samples can be extracted from the reconstructed consecutive holographic images. Therefore, the DIHM technique has been widely utilized for precise and quantitative measurement of the 3D behavioral characteristics exhibited by various microscale particles, including flow tracers in microfluidics [33–39], colloids [40–42], microbubbles [43, 44], particulate matter [45, 46], and microorganisms [47–50].

This review article presents an overview of the fundamental principles and applications of DIHM for quantitative analyses of cell dynamics in 3D volumes. The DIHM-based cell tracking procedures, including DIHM configuration, digital image preprocessing, numerical reconstruction, autofocusing, and particle tracking velocimetry (PTV) algorithms, are summarized. Recent studies on 3D dynamic analysis of living cells using DIHM technique are also discussed, covering a range of organisms such as erythrocytes, spermatozoa, bacteria, dinoflagellates, and algae. Experimental investigations into various dynamic behaviors of living cells, encompassing single-cell motilities, cell-cell interactions, and cell-surface interactions, are reviewed. Finally, recent studies on label-free sensing and classification of different types of living cells, such as diseased cells and microorganisms, demonstrate the potential clinical applications of DIHM technique for facile and accurate diagnosis of cellular diseases.

## Principles of DIHM

### Optical configuration of DIHM

**Fig. 1:**
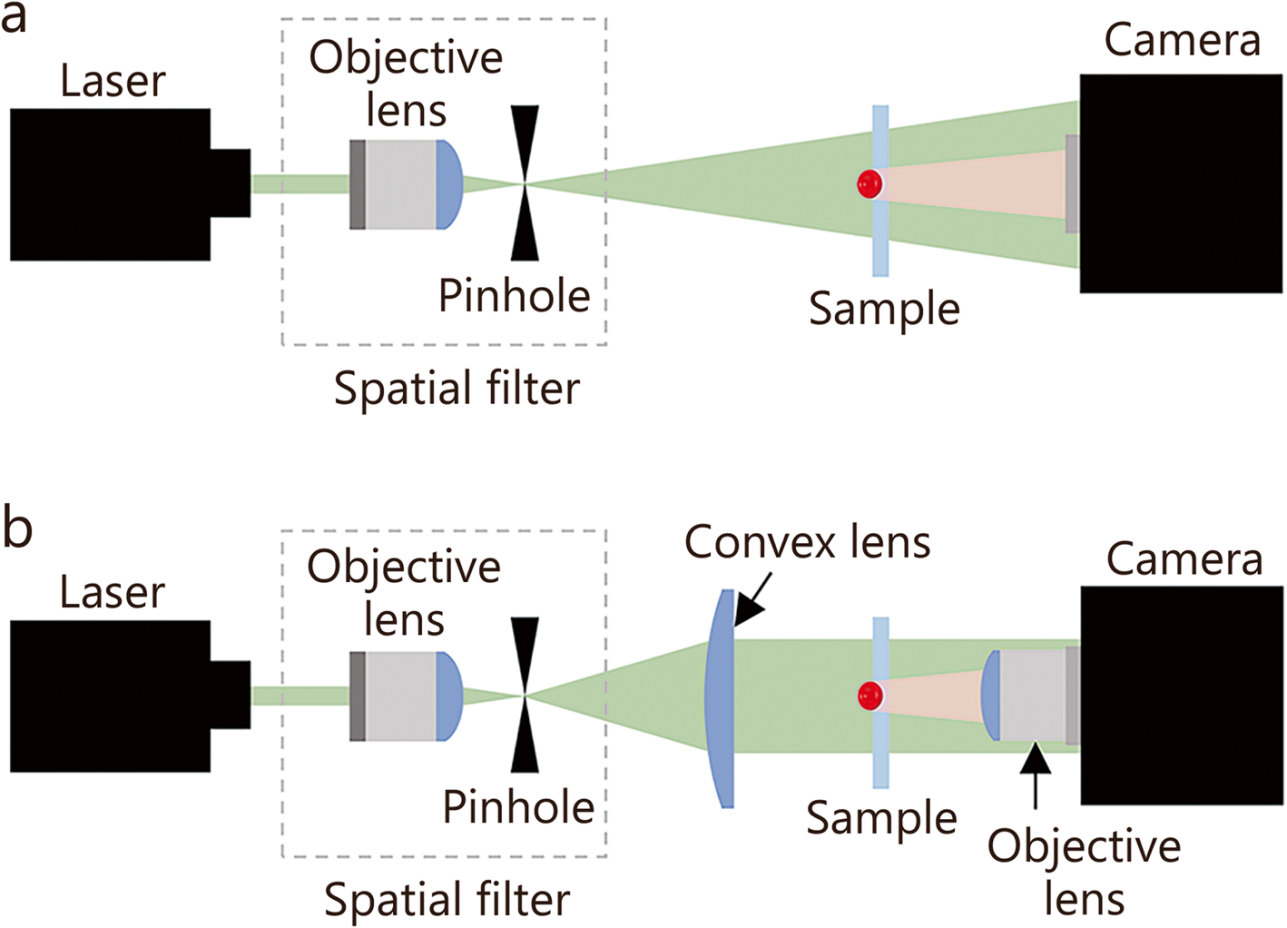
Schematics of the optical configurations of digital in-line holographic microscopy using a point source (**a**) and a collimated beam (**b**)

The basic configuration of DIHM, which uses a point light source, is derived from Gabor holography (Fig. 1a) [24]. It consists of a coherent laser source, a spatial filter, and a digital camera. A coherent laser beam with spherical waves is scattered from a test sample to generate an object beam. When the distance between the light source and the image sensor of the digital camera is sufficiently large, the incident wave can be approximated as a plane wave. A reference beam represents an unaffected wave emitted from the light source. In the DIHM configuration, the object and reference beams propagate in the same direction and interfere to form holographic interference patterns recorded on the image sensor. The magnification ratio can be adjusted by changing the ratio of the distance between the pinhole and the test sample to that between the pinhole and the camera. On the other hand, increasing the distance between the pinhole and the camera decreases the numerical aperture (NA) of the DIHM configuration using spherical waves. In addition, the magnification ratio and light intensity of holographic signals vary depending on the depth-wise position of the test sample. To visualize 3D dynamic behaviors of test samples over a wide volume with extended DOF, a collimated laser beam is utilized to configure the DIHM system (Fig. 1b) [29]. For this purpose, a convex lens converts spherical waves into plane waves, while an additional objective lens attached in front of the image sensor of the camera increases the magnification ratio. The holographic images captured by the DIHM system using plane waves maintain a constant pixel length and resolution, regardless of the depth position of the test sample. This advantage facilitates the design of experimental setups and enables the application of reconstruction algorithms to analyze 3D dynamic behaviors of test samples moving in a large volume. The field of view (FOV) is calculated by dividing the physical dimension of an image sensor by the magnification ratio of the DIHM system. The lateral and longitudinal resolutions are defined as |Δ*r*_lateral_|≥ 0.5*λ*/NA and |Δ*r*_longitudinal_|≥ 0.5*λ*/NA^2^, where *λ* represents the wavelength of the light source [26].

Several other in-line holographic configurations have recently been introduced. The DIHM configuration can be further simplified by replacing the coherent laser source with an inexpensive partially coherent light source, such as a light-emitting diode [32, 51–53]. Due to the lack of expensive objective lenses and a coherent laser source, the lensless DIHM system can be employed for developing compact portable devices to monitor various microscale particulates. Instead of using a coherent light source, spatially incoherent sunlight or fluorescence has recently been utilized for developing incoherent digital holography techniques, including optical scanning holography, Fresnel incoherent correlation holography, and coded aperture correlation holography [54–57]. To resolve the twin-image problem of DIHM configuration, single-shot in-line phase-shifting interferometry has been developed [58–60]. A phase-shifting array device is employed to capture multiple holograms with various phase shifts. This enables precise measurements of the phase information of a test object from the captured holograms and enhances the quality of reconstructed images. Multi-wavelength DIHM systems are utilized to extract multiple single-wavelength holograms from one single-shot hologram [61–64]. These extracted holograms are then used for phase unwrapping and comprehensive analysis of 3D morphological characteristics of biological samples illuminated with different wavelengths.

### Preprocessing of holographic images

Raw holographic images of a test sample contain unintended background noises induced by static dust particles and scratches on optical components. There are several methods available to remove these background noises and acquire clean holographic signals from the test sample. Firstly, the raw holographic images are normalized using the background image obtained by recording a holographic image without the test sample [65–67]. Secondly, normalization is performed based on the illumination intensity to effectively suppress multiplicative artifacts [40, 68]. Thirdly, spatially invariant background noises are subtracted from the raw holographic images by calculating an ensemble average of hundreds of consecutive holograms [29, 69]. Further improvement of the signal-to-noise ratio in holographic images can be achieved by employing various denoising techniques, such as band-pass filters [70–72], mean filters [73], median filters [74], Wiener filters [75], local-mean-subtraction filters [76], spectral filters [77], wavelet-based denoising [78], non-local means filtering [79], correlation-based denoising [80], and deep-learning-based denoising methods [81–83]. Super-resolution techniques can also be adopted to achieve high-resolution holographic imaging results [84–88].

### Numerical reconstruction of holographic images

Holographic images located at different depths away from the image plane can be numerically reconstructed by adopting several light diffraction theories [89, 90]. Kirchhoff-Helmholtz transform has been utilized to reconstruct holographic images (*H*_r_) from the original holographic image (*H*_o_) recorded by a DIHM setup with a point light source [91–93]. The reconstruction equation of the Kirchhoff-Helmholtz transform is expressed as follows:

1 $$H_{\mathrm r}(r)=\frac1{4\pi}\int_s{d\psi H_{\mathrm o}(\psi)}\exp\left[ik\psi\cdot\left(\frac{r}{|\psi|}\right)\right]\,$$

where *H*_o_(*ψ*) is the contrast image on the detector screen *S* at coordinates *ψ* = (*x*, *y*, *l*) located at a depth-wise distance *l* away from the point source. *r* and *k* denote the position vector from the point source and the wave number of the light source, respectively.

The Kirchhoff’s theory yields precise empirical results. However, the boundary conditions of Kirchhoff’s theory imply the absence of waves behind the aperture, leading to mathematical and physical contradictions [89]. To eliminate these inconsistencies associated with the boundary conditions in the Kirchhoff’s theory, the Rayleigh-Sommerfeld diffraction integral is utilized to reconstruct holograms of both spherical and plane waves [94–97]. The Rayleigh-Sommerfeld propagator (*h*), which enables wave field recovery, can be expressed as follows:

2 $$h = \frac{1}{2\pi }\frac{\partial }{\partial z}\frac{\exp (ikr)}{r} = \,\frac{1}{2\pi }\frac{\partial }{\partial z}\frac{{\exp \left[ {ik\sqrt {{{(\xi - x)}^2} + {{(\eta - y)}^2} + {z^2}} } \right]}}{{\sqrt {{{(\xi - x)}^2} + {{(\eta - y)}^2} + {z^2}} }}\,$$

where *x* and *y*, and *ξ* and *η* are the spatial coordinates on the image and reconstruction planes, respectively. *z* is the distance from the image plane to the reconstruction plane. The diffraction integral is expressed as follows:

3 $$H_{\mathrm r}(\xi,\eta;z)=\iint H_{\mathrm o}(x,y;0)\cdot h(\xi-x,\eta-y;z)dxdy$$

where *H*_r_(*ξ*,*η*;*z*) and *H*_o_(*x*,*y*;0) are the reconstructed and original holographic images, respectively.

Based on the Fresnel approximation, the diffraction integral can be converted into a simpler expression [27, 80, 98]. The hologram reconstruction process employing the Fresnel transformation can be expressed as follows:

4 $$H_{\mathrm r}(\xi,\eta;z)=\frac{\exp(ikz)}{i\lambda z}\iint H_{\mathrm o}(x,y;0)\cdot\exp\left\{\frac{ik}{2z}\left[{(\xi-x)}^2+{(\eta-y)}^2\right]\right\}dxdy\,$$

The Fresnel approximation exhibits precise reconstruction performance for small diffraction angles. The Fraunhofer approximation can be adopted to further simplify the transformation equation in the following manner:

5 $$H_{\mathrm r}(\xi,\eta;z)=\frac{\exp(ikz)\exp\left[\frac{ik}{2z}\left(\xi^2+\eta^2\right)\right]}{i\lambda z}\iint H_{\mathrm o}(x,y;0)\cdot\exp\left[-\frac{ik}z\left(\xi x+\eta y\right)\right]dxdy$$

where the quadratic terms of *x*^2^ + *y*^2^ are omitted [89]. The Fraunhofer transformation facilitates rapid calculation of the propagating wavefronts in far-field imaging.

Angular spectrum method is usually employed for reconstructing holographic images captured by a DIHM setup using plane waves [29, 99]. The angular spectrum of the wavefront recorded in the original holographic image is obtained through the application of fast Fourier transform (FFT), enabling extraction of the spatial frequency components distribution contained in the holographic image. Each spatial frequency component propagates through the space at different distances and angles. By applying the inverse FFT to these propagated spatial frequency components, a new hologram reconstructed at a depth-wise distance *z* from the original hologram can be obtained. This method does not require the minimum *z*-distance or any assumptions such as the Fresnel approximation, making it suitable for conducting 3D dynamic analyses of test samples moving in a large volume. The reconstruction equation of the angular spectrum method is expressed as follows:

6 $$H_{\mathrm r}(\xi,\eta;z)=F^{-1}\left\{F\left[H_{\mathrm o}(x,y;0)\right]\text{exp}\left[ikz\sqrt{1-\left(\lambda f_x\right)^2-\left(\lambda f_y\right)^2}\right]\right\}\,$$

where *F* and *F*^−1^ represent FFT and inverse FFT, respectively. *f*_x_ and *f*_y_ denote the spatial frequencies of *x* and *y* coordinates, respectively. To further improve the spatial resolution and signal-to-noise ratio of the reconstructed holograms, additional methodologies are adopted to suppress the twin-image problem associated with DIHM technique [61, 100–106].

### 3D localization of test samples using autofocusing algorithms

The 3D positional information of test samples is determined based on the reconstructed holograms, which are obtained by numerically reconstructing holograms at different depth-wise distances from an original hologram of a test sample. These reconstructed holograms are then projected into a single image plane. In-plane (*x*, *y*) position of each particle recorded on the projected hologram is determined by identifying extreme values in local intensity, image contrast, or sharpness. Among the reconstructed holograms of the object at the determined in-plane position, the degree of sharpness and image contrast (i.e., focus value) of the holograms is quantified by adopting various autofocusing functions. Subsequently, the depth-wise position *z* of each particle is determined by searching an extreme peak in focus values obtained by using focus functions, such as gradient (GRA), Laplacian (LAP), weighted spectral (SPEC), Tamura coefficient (TC), and variance (VAR) focus functions. These focus functions used for calculating focus values are defined as follows:

7 $$GRA(z) = \sum\limits_{\xi ,\eta } {\left| {\nabla {H_\text r}(\xi ,\eta ;z)} \right|}$$

8 $$LAP(z) = \sum\limits_{\xi ,\eta } {{{\left[ {{\nabla^2}{H_\text r}(\xi ,\eta ;z)} \right]}^2}}$$

9 $$SPEC(z)=\sum\limits_{f_\xi,f_\eta}\log\left\{1+\left|F\left[H_{\mathrm r}(\xi,\eta;z)-{\overline H}_\text{r}(z)\right]\right|\right\}$$

10 $$TC(z) = \sqrt {\frac{{\sigma \left[ {{H_\text r}(\xi ,\eta ;z)} \right]}}{{{{\overline H}_\text r}(z)}}}$$

11 $$VAR(z) = \frac{1}{{{N_\xi }{N_\eta }}}\sum\limits_{\xi ,\eta } {{{\left[ {{H_\text r}(\xi ,\eta ;z) - {{\overline H}_\text r}(z)} \right]}^2}}$$

where *σ* is the standard deviation [107, 108]. The resolution of the reconstructed hologram is *N*_*ξ*_ × *N*_*η*_ pixels. *f*_*ξ*_ and *f*_*η*_ denote the spatial frequencies of *ξ* and *η* coordinates, respectively. ${\overline H_r}(z)$ represents the spatial average value of a hologram reconstructed at the depth-wise position *z*. In the Rayleigh-Sommerfeld diffraction integral-based reconstruction process, the Gouy phase anomaly is utilized for the 3D localization of test samples [62, 97, 109]. Additionally, Gini’s index [110], Tenengrad function [111], Brenner function [112], DarkFocus algorithm [113], spectral *L*_1_ norm [114, 115], and novel deep-learning-based methodologies [116–120] are employed for autofocusing. Due to variations in experimental conditions such as the size and shape of test samples and the relative refractive index of media, it is important to consider an appropriate autofocusing method for a given experimental condition.

### 3D PTV algorithms for cell tracking

The 3D dynamic behaviors of test samples can be analyzed by extracting the 3D positional information from reconstructed consecutive holographic images. Furthermore, the trajectory of test samples can be easily obtained by connecting the 3D positions of individual objects in subsequent holograms. Several 3D PTV algorithms can be applied to thousands of 3D positional information of test samples to obtain their trajectories. For example, the two-frame PTV algorithm based on iterative estimation of match probability can be utilized for rapid tracking of particle trajectory using only two successive image frames [121]. The Crocker-Grier algorithm can be employed to search for the probable set of particle indices and locations in each frame among the successive and preceding image frames, taking into account various factors such as size, intensity, and displacement of particles to ensure accurate tracking [122, 123]. Additionally, high-order multi-frame tracking algorithms are employed for robust and accurate 3D Lagrangian tracking of particles [124–128]. Machine learning-based cell tracking algorithms can also be adopted to improve the performance of holographic PTV measurements. A neural network is used for nonlinear global regression to filter out random noises present in PTV data and reconstruct the entire flow field from captured photographs [129]. To overcome technical limitations associated with previous PTV algorithms when dealing with highly concentrated tracer particles and high-speed flows, a long short-term memory network is utilized to predict the subsequent velocity of a tracer particle based on its past PTV data [130].

### Alternative holographic processing techniques

Conventional digital image processing methods for holographic PTV typically include preprocessing, numerical reconstruction, autofocusing, and particle tracking. Recently, several alternative holographic processing techniques deviating from traditional categories have been proposed. For example, 3D volumetric deconvolution method utilizes a point-spread function to enhance the optical features contained in reconstructed holograms [131–133]. It effectively resolves the superimposed out-of-focus signals of highly concentrated particles. Additionally, various inverse reconstruction methods employing fused lasso regularization [134], Tikhonov regularization [135], and iterative predictive algorithm [136] are introduced to overcome the technical limitations of DIHM in terms of particle concentration and reconstruction dynamic range.

Recently, artificial intelligence (AI) has been applied to expedite the computational time required for numerical reconstruction and autofocusing procedures in digital image processing. Specifically, a convolutional neural network (CNN) is trained using holograms of test samples and corresponding ground-truth depth-wise position labels [118]. By leveraging this trained CNN model, it can directly predict the depth-wise positions of test samples from their holograms without resorting to numerical reconstruction and autofocusing procedures. To achieve this, a CNN architecture composed of convolutional layers, up-sampling blocks, and nonlinear activation functions is trained with pairs of raw holograms and their corresponding reconstructed amplitude and phase maps [104]. Additionally, a U-Net architecture is utilized to predict 3D locations of highly concentrated particles based on input holograms, depth maps, and maximum phase projections [137]. Furthermore, a fusion approach involving two U-Nets is trained with raw holograms at the input layer of one down-sampling path and pairs of intensity and phase maps at the output layer of two up-sampling paths [138]. Moreover, a generative adversarial network, which utilizes mutual training of the generator and discriminator, is employed to generate in-focus intensity and phase maps from an input hologram [139–141]. In addition to these techniques mentioned above, a Fourier imager network consisting of spatial Fourier transform modules can provide a global receptive field for processing holographic diffraction patterns obtained from test samples [142]. Lastly, a self-supervised learning model is trained by using a physics-consistency loss along with synthetic images instead of generating experimental datasets [143]. Therefore, recent advancements in AI techniques facilitate rapid hologram reconstruction and precise localization in cell tracking, thereby replacing the time-consuming digital image processing procedures of traditional DIHM methods.

## Applications of DIHM to various microscale biological cells

### 4D tracking of human blood cells

**Fig. 2:**
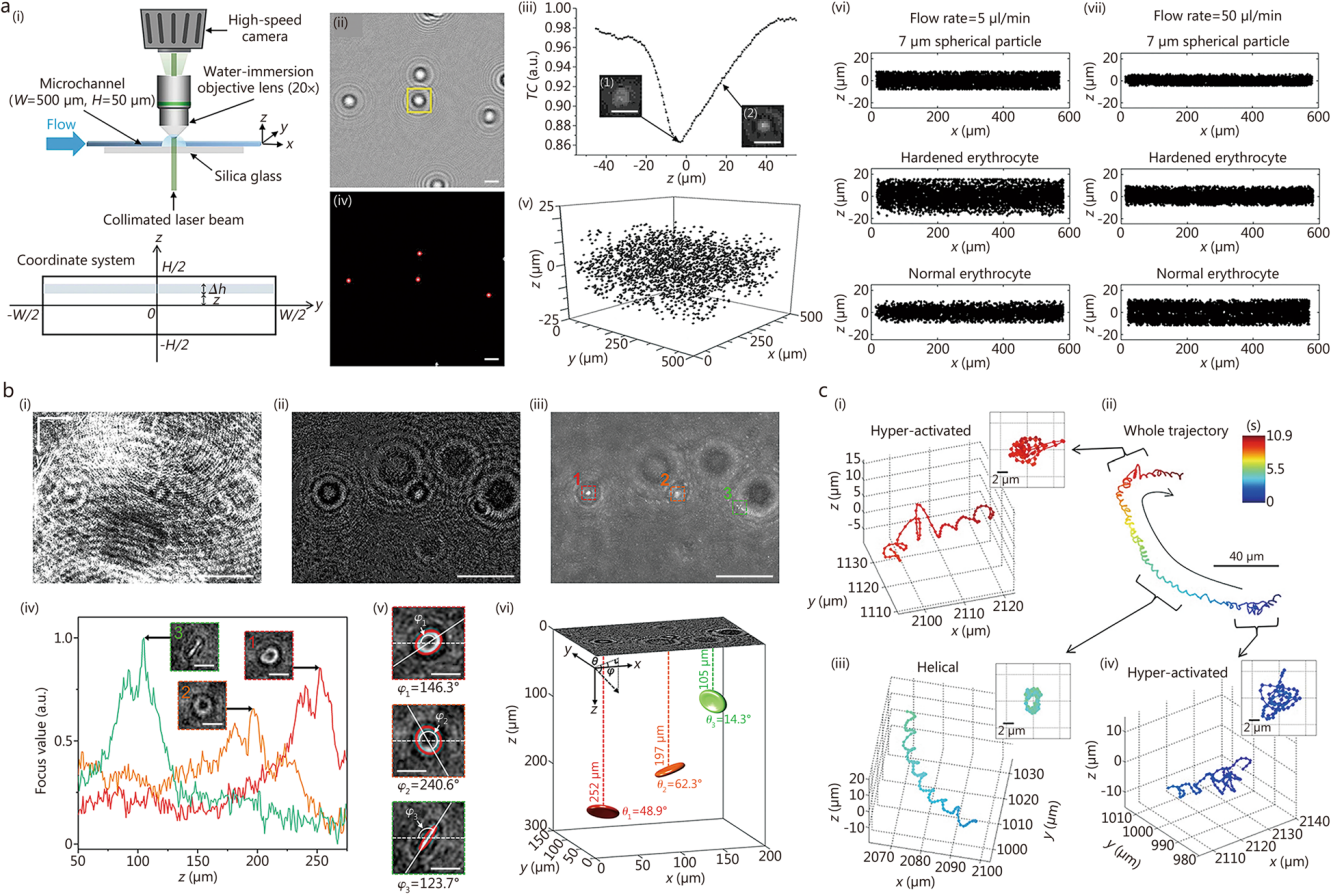
Applications of digital in-line holographic microscopy (DIHM) to track human erythrocytes and spermatozoa. **a** Lateral migration of hardened and normal erythrocytes in viscoelastic flows under different microfluidic conditions. Experimental setup for the microfluidic measurement (i). Digital image processing procedure: background subtraction (ii, scale bar = 10 μm), depth localization using a Tamura coefficient (TC) focus function (iii, scale bar = 10 μm), in-plane positioning (iv, scale bar = 10 μm), and 3D spatial distributions of spherical particles, hardened erythrocytes, and normal erythrocytes measured using DIHM (v-vii). Reprinted from ref. [149], Copyright 2017. **b** Measurement of 3D locations and orientations of erythrocytes using DIHM and deep learning techniques. Digital image processing procedure: raw hologram (i, scale bar = 20 μm), background subtraction (ii, scale bar = 20 μm), projection (iii, scale bar = 20 μm), depth localization using a gradient focus function (iv, scale bar = 5 μm), in-plane angle measurement (v, scale bar = 5 μm), and 3D positions and orientations of erythrocytes measured using DIHM (vi). Reprinted with permission from ref. [151], Copyright 2023, Elsevier B.V. **c** Transitions between different swimming patterns of a human spermatozoon. Hyper-activated (i, iv) and helical patterns (iii) are observed in a whole trajectory of the human spermatozoon (ii). Reprinted from ref. [152], Copyright 2012

The DIHM technique enables effective analysis of 4D dynamic behaviors of human blood cells, including neutrophils and erythrocytes (Table 1) [144–151]. For example, rapid movement of neutrophils (HL60 cells) was visualized with lateral and longitudinal resolutions in the range of a few micrometers [144]. A DIHM system was employed to track cell migration of unlabeled asthmatic and non-asthmatic neutrophils at a high temporal resolution [145]. Their study comparatively investigated the averages of undirected speed and outward velocity of neutrophils exposed to various chemotactic stimuli (interleukin-8 and N-formylmethionyl-leucyl-phenylalanine) under different mechanical environments such as collagen stiffness and pore size. The 3D velocity profile of erythrocytes in Hagen-Poiseuille flows was measured depending on their radial position while flowing through a microtube with an inner diameter of 350 μm [146]. Statistical analysis was conducted on the inertial migration phenomena exhibited by erythrocytes moving in cylindrical and rectangular microchannels under various microfluidic conditions [147, 148]. 3D spatial distributions of erythrocytes and microspheres were investigated under different shear rates. Quantitative comparisons were made between healthy and hardened erythrocytes, regarding their lateral migration, deformation index, and orientation for various flow rates of viscoelastic fluids in a rectangular microchannel (Fig. 2a) [149]. The typical experimental setup for microfluidic DIHM measurements is illustrated in Fig. 2ai. Digital image processing procedures involving background subtraction (Fig. 2aii), depth localization using a TC focus function (Fig. 2aiii), as well as in-plane positioning (Fig. 2aiv), were utilized to detect the 3D spatial distribution of erythrocytes. The spatial distributions of spherical particles, hardened erythrocytes, and normal erythrocytes in microchannels were obtained with varying flow rates in microchannels (Fig. 2av-vii).

**Table 1:** Summary of previous studies on four-dimensional (4D) tracking of human blood cells using digital in-line holographic microscopy

Year	Object	Content	Reconstruction method	Axial localization algorithm	Tracking algorithm	References
2008	Neutrophil	Three-dimensional (3D) dynamic behavior of fast-moving neutrophils in suspension cultures	Wavelet transform derived from Fresnel transformation	Local extreme intensity	Connecting 3D positions for each object in subsequent holograms	[144]
2022	Neutrophil	Comparison of cell migration of asthmatic and non-asthmatic neutrophils subjected to different chemotactic stimuli and mechanical environments	Angular spectrum method	Thresholding minimum projections	Crocker-Grier algorithm	[145]
2009	Erythrocyte	The 3D motion of erythrocytes in Hagen-Poiseuille flows in microtubes	Angular spectrum method	Laplacian (LAP) focus function	Two-frame particle tracking velocimetry (PTV) algorithm	[146]
2012	Erythrocyte	Inertial migration of erythrocytes in low-viscosity and high-shear rate microtube flows	Angular spectrum method	Determining based on the velocity profile of a Poiseuille flow	Crocker-Grier algorithm	[147]
2014	Erythrocyte	Inertial migration of erythrocytes in water and viscoelastic flows in rectangular microchannels	Angular spectrum method	LAP focus function	Superimposing 3D positions of objects in subsequent holograms	[148]
2017	Erythrocyte	Comparison of inertial migration, deformation index, and orientation of normal and hardened erythrocytes in viscoelastic flows in rectangular microchannels	Angular spectrum method	Tamura coefficient focus function	Superimposing 3D positions of objects in subsequent holograms	[149]
2018	Erythrocyte	Digital stereo-holographic microscopy for measuring 3D position, orientation, and morphology of erythrocytes	Angular spectrum method	Intensity thresholding	Two-frame PTV algorithm	[150]
2023	Erythrocyte	Deep-learning-based measurement of 3D position and orientation of erythrocytes	Angular spectrum method	Gradient focus function	No tracking	[151]

Stereo-holographic microscopy was developed to obtain 3D positional, orientational, and morphological information on non-spherical particles, including ellipsoidal particles and erythrocytes [150]. To accurately measure the 3D orientations of erythrocytes, a deep-learning-based DIHM technique was developed, which can estimate the in-plane and out-of-plane angles of erythrocytes from their in-focus reconstructed holograms (Fig. 2b) [151]. The signal-to-noise ratio of holographic signals of three erythrocytes recorded in a raw hologram (Fig. 2bi) was enhanced by subtracting background noises **(**Fig. 2bii). The reconstructed holograms of erythrocytes were projected on a single image plane to determine in-plane locations of erythrocytes by searching for local intensity extrema (Fig. 2biii). Depth locations and in-focus reconstructed holograms of erythrocytes were obtained by employing a GRA focus function (Fig. 2biv). The in-plane angle (*φ*) of each erythrocyte was determined by evaluating the inclined angle of its major axis (Fig. 2bv). A convolutional autoencoder was utilized to increase the number of holographic images with respect to the out-of-plane angle of erythrocytes. Subsequently, a CNN for regression analysis was used to directly predict the out-of-plane angle (*θ*) from the reconstructed hologram of each erythrocyte (Fig. 2bvi). On the other hand, previous studies on erythrocytes have mainly focused on measuring their 3D translational dynamics in straight microchannels. Further development is required for DIHM technique with the aid of AI to analyze the 3D rotational dynamics exhibited by moving erythrocytes within more complex microfluidic conduits.

### 4D tracking of spermatozoa

The swimming motility of human and animal spermatozoa can be analyzed by visualizing 3D trajectories of spermatozoa swimming in a 3D volume using a DIHM technique (Table 2) [152–157]. For example, submicron accuracy was achieved in tracking various swimming patterns of human spermatozoa, including typical, helical, hyper-activated, and hyper-helical patterns [152]. The transitions between swimming patterns within each sperm’s trajectory were statistically investigated using a lens-free imaging platform (Fig. 2c). A whole trajectory of a human spermatozoon (Fig. 2cii) includes transitions from the hyper-activated pattern (Fig. 2civ) to the helical pattern (Fig. 2ciii), and back to the hyper-activated pattern again (Fig. 2ci). By adopting the lensless DIHM configuration, a cost-effective microscopic device can be established for analyzing the swimming motions of human spermatozoa with high resolution and a large FOV [153]. Spiral trajectories of goat spermatozoa exhibited significantly higher concentration and intense motility compared to those of human spermatozoa [154]. Quantitative visualization was performed on the 3D trajectories of *Arbacia punctulata* spermatozoa which navigate through 3D chemoattractant gradients provided by an egg for fertilization, revealing their tracking process toward the egg [155]. The swimming motions of horse spermatozoa were categorized into six different patterns, including irregular, linear, planar, helical, ribbon, and hyper-progressive patterns [156]. Free-swimming spermatozoa of normal and unhealthy mice were also comparatively investigated [157]. A double-knockout mouse model lacking tubulin glycosylation was generated by targeting the initiating glycylases tubulin-tyrosine ligase-like (TTLL) 3 and TTLL8 (*Ttll*3^−/−^*Ttll*8^−/−^ mouse). The DIHM technique was employed to measure the 3D behaviors of spermatozoa from both a normal mouse and a *Ttll*3^−/−^*Ttll*8^−/−^ mouse. The spermatozoa from the normal mouse exhibited twisted ribbon patterns, while those from the *Ttll*3^−/−^*Ttll*8^−/−^ mouse displayed helical patterns. As the spermatozoa from the *Ttll*3^−/−^*Ttll*8^−/−^ mouse approached the wall of the observation chamber, their swimming patterns transitioned from helical to circular motion. Consequently, this kind of change in swimming patterns disrupted their progressive movement. These experimental studies show that the DIHM technique possesses sufficient resolution and measurement accuracy for assessing the 3D swimming motions of spermatozoa.

**Table 2:** Summary of previous studies on four-dimensional (4D) tracking of spermatozoa using digital in-line holographic microscopy

Year	Object	Content	Reconstruction method	Axial localization algorithm	Tracking algorithm	References
2012	Human spermatozoon	Swimming patterns of spermatozoa, such as typical, helical, hyper-activated, and hyper-helical patterns	Weighted back-projection method	Local extreme contrast	Linking up the nearest three-dimensional (3D) positions of objects in subsequent holograms	[152]
2022	Human spermatozoon	Swimming motions of human spermatozoa	Angular spectrum method	DarkFocus algorithm	Connecting 3D positions for each object in subsequent holograms	[153]
2022	Human and goat spermatozoa	Comparison of swimming motions of human and goat spermatozoa	Angular spectrum method	DarkFocus algorithm	Connecting 3D positions for each object in subsequent holograms	[154]
2015	*Arbacia punctulata* (*A. punctulata*) spermatozoon	Swimming motions of *A. punctulata* spermatozoa navigating 3D chemoattractant gradients provided by an egg for fertilization	Rayleigh-Sommerfeld back-propagation	Gouy phase anomaly and Sobel filtering	Linking 3D positions into continuous trajectories using a custom-made tracking program written in Java	[155]
2016	Horse spermatozoon	Swimming patterns of horse spermatozoa, such as irregular, linear, planar, helical, ribbon, and hyper-progressive patterns	Weighted back-projection method	Local extreme contrast	Linking up the nearest 3D positions of objects in subsequent holograms	[156]
2021	Mouse spermatozoon	Comparison of swimming motions of a normal mouse and a mouse without tubulin glycosylation	Rayleigh-Sommerfeld back-propagation	Gouy phase anomaly	Connecting 3D positions for each object in subsequent holograms	[157]

### 4D tracking of bacteria

**Fig. 3:**
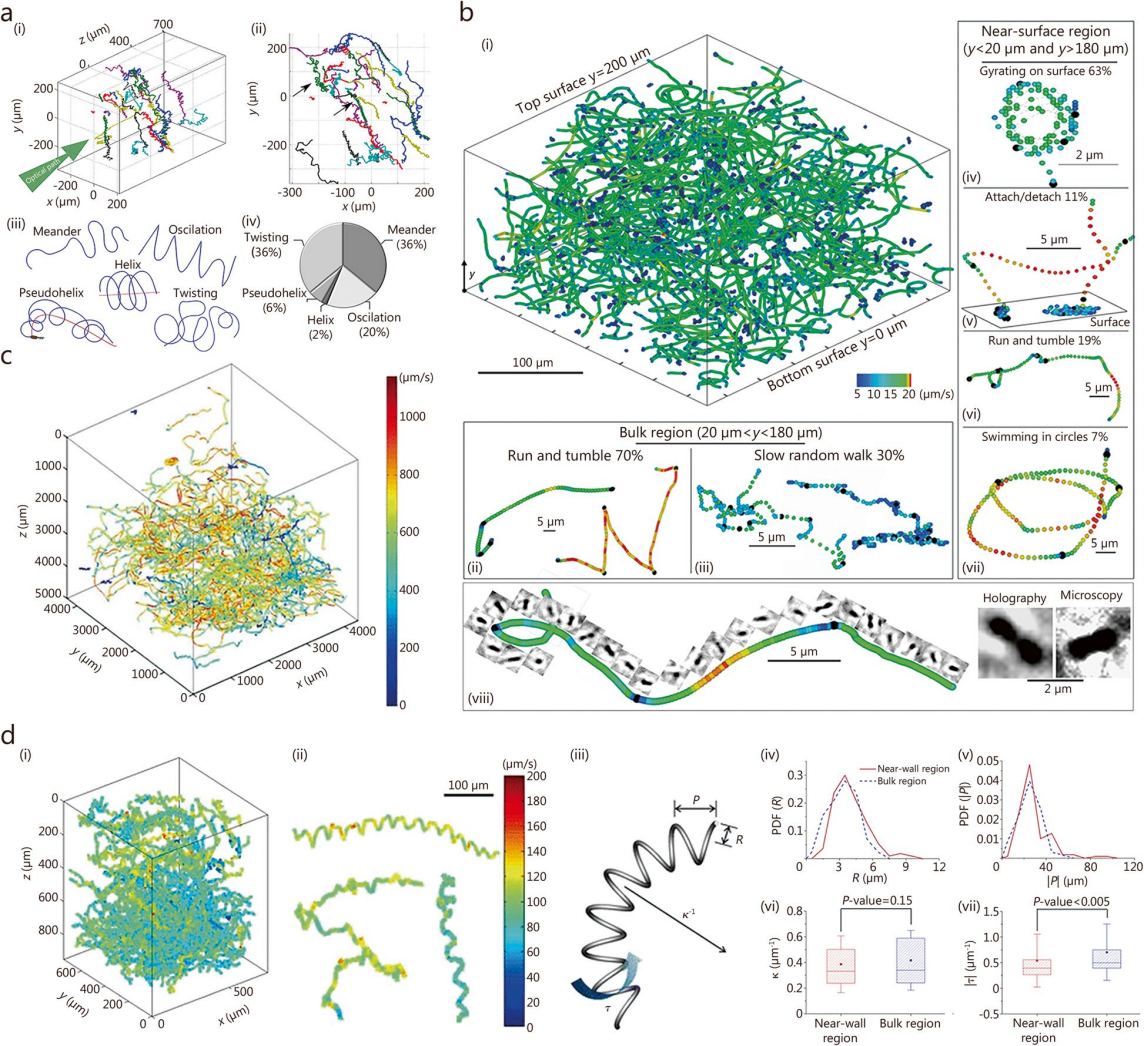
Applications of digital in-line holographic microscopy to track various unicellular microorganisms. **a** Trajectories of swimming *Pseudomonas aeruginosa* obtained by using DIHM (i, ii) and the corresponding statistical analysis of various swimming patterns, including meander, oscillation, helix, pseudohelix, and twisting patterns (iii, iv). Reprinted from ref. [158], Copyright 2014. **b** Various swimming patterns of *Escherichia coli* (*E. coli*) in near-surface and bulk regions. Trajectories of swimming *E. coli* (i, viii). Swimming patterns of *E. coli* in the bulk region: running and tumbling motions (ii) and slow random walk (iii). Swimming patterns of *E. coli* in the near-surface region: gyrating on a surface (iv), attaching and detaching motions (v), running and tumbling motions (vi), and swimming in circles (vii). Reprinted with permission from ref. [163], Copyright 2014, American Physical Society. **c** 3D trajectories of solitary and chain-forming *Cochlodinium polykrikoides*. Reprinted with permission from ref. [172], Copyright 2010, Springer-Verlag. **d** Trajectories of *Prorocentrum minimum* in helical motions (i-iii), obtained using DIHM. Probability density functions (PDFs) of helix parameters in the near and bulk regions: radius (*R*, iv) and pitch (*P*, v). Statistical differences in helix parameters between the near and bulk regions, represented as probability values (*P*-values): curvature (*κ*, vi) and torsion (*τ*, vii). Reprinted with permission from ref. [173], Copyright 2016, Springer-Verlag Berlin Heidelberg

The free-swimming behaviors of various bacteria have been quantitatively analyzed using a DIHM technique to reveal the underlying mechanisms of microbial motility (Table 3) [62, 80, 158–171]. *Pseudomonas aeruginosa* (*P. aeruginosa*) exhibited several swimming behaviors, including meandering, oscillation, helix, pseudohelix, and twisting patterns, and the transitions between different patterns were analyzed (Fig. 3a) [158]. Statistical comparisons were made on the 3D swimming speed and turning angle distributions of *P. aeruginosa*, *Agrobacterium tumefaciens*, and *Escherichia coli* (*E. coli*) [159]. Submicron-scale kinematics, morphological shape, and orientation measurements were conducted on individual *E. coli* to assess their motilities, including swimming speed, tumbling motion, and wobbling motion [62, 80, 160]. The body-angle rotation during runs, tumbles, and pole reversal in *E. coli* was measured using a C-implemented discrete dipole approximation code and the Levenberg-Marquardt algorithm [161]. The filament compositions of *Shewanella putrefaciens* were observed to affect the flagellar morphology and free-swimming trajectories [162].

**Table 3:** Summary of previous studies on four-dimensional (4D) tracking of bacteria using digital in-line holographic microscopy

Year	Object	Content	Reconstruction method	Axial localization algorithm	Tracking algorithm	References
2014	*Pseudomonas aeruginosa* (*P. aeruginosa*)	Swimming motility of *P. aeruginosa*, including meander, oscillation, helix, pseudohelix, and twisting patterns	Kirchhoff-Helmholtz transformation	Local extreme intensity	Satisfying self-consistency between trajectories projected on *xz*- and *yz*-planes	[158]
2015	*P. aeruginosa*, *Agrobacterium tumefaciens* (*A. tumefaciens*), and *Escherichia coli* (*E. coli*)	Swimming motility of *P. aeruginosa*, *A. tumefaciens*, and *E. coli*	Rayleigh-Sommerfeld back-propagation	Intensity thresholding	Connecting three-dimensional (3D) positions for each object in subsequent holograms	[159]
2014	*E. coli*	Swimming motility of *E. coli*	Fresnel transformation	Local extreme intensity	3D Lagrangian tracking algorithm	[80]
2016	*E. coli*	Swimming motility of *E. coli*., such as body angle rotation during runs, tumbles, and pole reversal	Discrete dipole approximation and Levenberg-Marquardt algorithm	Minimum sum of squared differences between the simulated model and measured holograms	Connecting 3D positions for each object in subsequent holograms	[161]
2017	*E. coli*	Swimming motility of *E. coli*	Rayleigh-Sommerfeld back-propagation	Local extreme intensity	Connecting 3D positions for each object in subsequent holograms	[62]
2023	*E. coli*	Swimming motility of *E. coli*	Angular spectrum method	Variance focus function	Superimposing 3D positions of objects in subsequent holograms	[160]
2018	*Shewanella putrefaciens* (*S. putrefaciens*)	Swimming motility of *S. putrefaciens* with varying filament compositions	Rayleigh-Sommerfeld back-propagation	Gouy phase anomaly and Sobel filtering	Connecting 3D positions for each object in subsequent holograms	[162]
2014	*E. coli*	Cell-surface interaction; Swimming motions of *E. coli* in near-surface and bulk regions, including gyrating on the surface, attaching, detaching, running, tumbling, swimming in circles, and slow random walk	Fresnel transformation	Local extreme intensity	3D Lagrangian tracking algorithm	[163]
2016	*E. coli*	Cell-surface interaction; Swimming motions of *E. coli* in a near-surface region under various flow shear	Fresnel transformation	Local extreme intensity	Frame-to-frame particle tracking velocimetry algorithm	[164]
2017	*E. coli*	Cell-surface interaction; Landing dynamics of *E. coli* near polymeric surfaces with varying surface hydrophobicity	Rayleigh-Sommerfeld back-propagation	Local extreme intensity	Linking 3D positions into continuous trajectories using home-made Python code	[165]
2017	*E. coli*	Cell-surface interaction; Swimming motions of *E. coli* during wall entrapment	Rayleigh-Sommerfeld back-propagation	Local extreme intensity	Connecting 3D positions for each object in subsequent holograms	[166]
2017	*E. coli* and *Pseudomonas* species	Cell-surface interaction; Swimming motions of *E. coli* and *Pseudomonas* species on biodegradable polymeric surfaces	Rayleigh-Sommerfeld back-propagation	Local extreme intensity	Linking 3D positions into continuous trajectories using home-made Python code	[167]
2019	*E. coli* and *Pseudomonas* species	Cell-surface interaction; Swimming motions of *E. coli* and *Pseudomonas* species on polymeric surfaces with varying surface stiffness	Rayleigh-Sommerfeld back-propagation	Local extreme intensity	Linking 3D positions into continuous trajectories using home-made Python code	[168]
2019	*P. aeruginosa*	Cell-surface interaction; Swimming motions of *P. aeruginosa* in a near-surface region	Rayleigh-Sommerfeld back-propagation	Intensity thresholding	Connecting 3D positions for each object in subsequent holograms	[169]
2023	*Shewanella* species	Cell-surface interaction; Swimming motions of *Shewanella* species in near-surface and bulk regions	Rayleigh-Sommerfeld back-propagation	Intensity thresholding	A three-frame predictive particle tracking algorithm	[170]
2023	*Enterobacter sakazakii* (*E. sakazakii*)	Cell-surface interaction; Swimming motions of *E. sakazakii* near the surfaces coated with sessile probiotics	Rayleigh-Sommerfeld back-propagation	Local extreme intensity	Linking 3D positions into continuous trajectories using home-made Python code	[171]

Quantitative measurement of bacteria cell-surface interactions is essential to understand diverse microbial behaviors, such as bacterial adhesion and biofilm formation, and the development of biomedical and antibiofouling surfaces. Various swimming motions of *E. coli* were observed in both near-surface and bulk regions (Fig. 3bi, viii), encompassing running and tumbling (Fig. 3bii, vi), slow random walk (Fig. 3biii), gyrating on a surface (Fig. 3biv), attaching and detaching (Fig. 3bv), and circular swimming (Fig. 3bvii) [163]. In the near-surface region, tumbling motions of *E. coli* were reduced by 50%, while reorientations were restricted to surface-parallel directions, impeding their escape from this region. Shear flow generated in the near-surface area promoted tumbling and reorientation movements, thereby enhancing bacterial dispersion [164]. Surface hydrophobicity reduced the swimming speed of *E. coli* in the near-surface region, promoting their landing on and adhesion to the surface [165].

The wall entrapment mechanism of *E. coli* was investigated by quantifying various parameters such as cell axis ratio, vertical speed, collision angle, pitch angle, and wobbling angle [166]. 3D motion analysis of *E. coli* and *Pseudomonas* species over biodegradable poly(*ε*-caprolactone)-based polymers revealed that enzymatic degradation rate was inversely correlated with irreversible adhesion [167]. Similarly, decreasing surface stiffness led to reduced bacterial adhesion for *E. coli* and *Pseudomonas* species on polydimethylsiloxane surfaces [168]. Swimming behaviors were found to differ between wild-type strains and isogenic flagellar stator mutants of *P. aeruginosa* in near-surface environments [169]. *Shewanella* species exhibited faster swimming speeds and longer trajectories in the bulk region, despite accumulating in the region near the surface [170]. *Enterobacter sakazakii* (*E. sakazakii*) showed adaptive swimming behaviors in the region near the surface coated with sessile probiotics, reducing wall accumulation [171].

### 4D tracking of dinoflagellates

Swimming speeds of *Alexandrium ostenfeldii*, *Alexandrium minutum*, and *Alexandrium tamarense* were compared at different temperatures [174]. Prey-induced changes in the swimming behaviors of *Karlodinium veneficum* (*K. veneficum*) and *Pfiesteria piscicida* were compared in terms of the radius and pitch of their helical swimming trajectories, as well as their translational and angular velocities [98]. Quantitative visualization was performed on the 3D trajectories of predatory *K. veneficum* and prey *Storeatula major* immobilized by karlotoxins [175]. Using a DIHM technique, the helical swimming trajectories of *Cochlodinium polykrikoides* (*C. polykrikoides*) and *Prorocentrum minimum* (*P. minimum*) were also analyzed [72]. The motile characteristics of solitary cells vs. chain-forming cells showed that the swimming speed, helix radius, and pitch of 3D trajectories increased with an increasing number of cells in the *C. polykrikoides* chain (Fig. 3c) [172]. As the viscosity of the surrounding media increased, both the swimming speed and flagella beating frequency decreased for *P. minimum* [176]. The hydrodynamic power consumed for the swimming motion of *P. minimum* was quantitatively estimated. The analyzed 3D swimming trajectories and helix parameters indicated that motility and thrust generation were higher in the near-surface region for *P. minimum* (Fig. 3d) [173]. Based on the measured 3D trajectories of *P. minimum* showing helical motions (Fig. 3di-iii), the basic helix parameters, including radius (*R*, Fig. 3div), pitch (*P*, Fig. 3dv), curvature (*κ*, Fig. 3dvi), and torsion (*τ*, Fig. 3dvii), were evaluated in the near and bulk regions. An abrupt drifting motion exhibited by *P. minimum* was tracked using a lens-free configuration [177]. Previous studies on the 4D tracking of dinoflagellates are summarized in Table 4 [72, 98, 172–177].

**Table 4:** Summary of previous studies on four-dimensional (4D) tracking of dinoflagellates using digital in-line holographic microscopy

Year	Object	Content	Reconstruction method	Axial localization algorithm	Tracking algorithm	References
2006	*Alexandrium* species	Swimming motility of *Alexandrium* species	Kirchhoff-Helmholtz transformation	Local extreme sharpness and intensity	Superimposing in-focus reconstructed holograms	[174]
2007	*Karlodinium veneficum* (*K. veneficum*) and *Pfiesteria piscicida* (*P. piscicida*)	Prey-induced change in swimming behavior of *K. veneficum* and* P. piscicida*	Fresnel transformation	Local extreme sharpness and intensity	Three-dimensional (3D) Lagrangian tracking algorithm	[98]
2010	*K. veneficum*	Swimming motility of *K. veneficum* and *Storeatula major*	Fresnel transformation	Local extreme sharpness and intensity	3D Lagrangian tracking algorithm	[175]
2011	*Cochlodinium polykrikoides* (*C. polykrikoides*) and *Prorocentrum minimum* (*P. minimum*)	Swimming motility of *C. polykrikoides* and *P. minimum*	Angular spectrum method	Laplacian and Variance (VAR) focus functions	Two-frame particle tracking velocimetry (PTV) algorithm	[72]
2011	*C. polykrikoides*	Swimming motility of solitary and chain-forming *C. polykrikoides*	Angular spectrum method	VAR focus function	Two-frame PTV algorithm	[172]
2013	*P. minimum*	Swimming motility of *P. minimum* according to medium viscosity	Angular spectrum method	VAR focus function	Two-frame PTV algorithm	[176]
2016	*P. minimum*	Swimming motility of *P. minimum* in near-surface and bulk regions	Angular spectrum method	VAR focus function	Two-frame PTV algorithm	[173]
2021	*P. minimum*	Swimming motility of *P. minimum*	Angular spectrum method	Local extreme sharpness	Connecting 3D positions for each object in subsequent holograms	[177]

### 4D tracking of other biological cells

3D trajectories of free-swimming algae *Tetraselmis* species in seawater were measured using a DIHM technique [178]. Unknown species of algae and bacteria were collected from glacial meltwater and investigated using DIHM in terrestrial and exobiological studies [179]. The relationship between inertial migration and elastic shell compliance was analyzed by measuring the spatial distributions of normal and hardened *Chlorella* cells in a pipe flow [180]. 3D swimming trajectories of *Dunaliella primolecta* (*D. primolecta*) in shear flow were measured to investigate the shear-induced algal migration, compared to those in a quiescent fluid [181]. Complex helical trajectories and velocity fluctuations of free-swimming *D. primolecta* across different growth phases were also statistically measured using the DIHM technique [134, 182]. *Ulva* zoospores exhibited various swimming patterns in the near-surface and bulk regions, including straight path, gyration, search circle, orientation, and wobbling motions [183]. Swimming velocity and diving direction of *Ulva* zoospores near glass surfaces were statistically analyzed [184]. Additionally, 3D trajectories of *Ulva* zoospores were monitored over various surfaces, such as polyethylene glycol coating, acid-washed glass, and tridecafluorooctyl-triethoxysilane-coated glass surfaces [185].

Underwater DIHM was utilized to observe a diverse range of microorganisms in oceans or lakes, such as *Paramecium* species, *Ciliate* species, *Didinium* species, and *Coscinodiscus wailesii* (diatom) [26]. The 3D motility of blood-parasite *Trypanosoma brucei* was quantified under various external conditions to investigate the mechanism of immune evasion [186]. Optimal chemotactic behaviors of *Haloarchaea* (*Haloferax* species and *Haloarcula* species) were examined to reveal the survival strategies of archaea under extreme, nutrient-poor conditions [187]. The 3D trajectory analysis unveiled the active diving motion exhibited by a neuroblastoma cell [177]. Infective and non-infective intracellular parasites (*Leishmania mexicana*) displayed distinctive swimming patterns and chemotaxis towards human cells [188]. Furthermore, DIHM technique was utilized for measuring size distributions and trajectories of airborne virus-laden droplets and aerosols, such as MS2 bacteriophage and severe acute respiratory syndrome coronavirus 2 [189]. These previous studies underscore the immense potential of DIHM technique for in situ, real-time, and non-invasive measurements of 3D dynamic behaviors across various biological cells. A summary table containing these studies on 4D tracking is presented in Table 5 [26, 134, 177–189].

**Table 5:** Summary of previous studies on four-dimensional (4D) tracking of other biological cells using digital in-line holographic microscopy

Year	Object	Content	Reconstruction method	Axial localization algorithm	Tracking algorithm	References
2003	*Tetraselmis* species	Swimming motility of *Tetraselmis* species	Kirchhoff-Helmholtz transformation	Local extreme sharpness and intensity	Superimposing in-focus reconstructed holograms	[178]
2010	Unknown algae and bacteria	Swimming motility of unknown living organisms	Kirchhoff-Helmholtz transformation	Local extreme sharpness and intensity	Superimposing in-focus reconstructed holograms	[179]
2014	*Chlorella*	Inertial migration of normal and hardened *Chlorella* cells in a pipe flow	Angular spectrum method	Variance focus function	Superimposing three-dimensional (3D) positions of objects in subsequent holograms	[180]
2013	*Dunaliella primolecta* (*D. primolecta*)	Swimming motility of *D. primolecta* under different fluidic conditions	Fresnel transformation	Local extreme sharpness and intensity	3D Lagrangian tracking algorithm	[181]
2019	*D. primolecta*	Swimming motility of *D. primolecta*	Rayleigh-Sommerfeld back-propagation	Fast iterative shrinkage-thresholding algorithm	Crocker-Grier algorithm	[134]
2020	*D. primolecta*	Swimming motility of *D. primolecta* across growth phases	Rayleigh-Sommerfeld back-propagation	Fast iterative shrinkage-thresholding algorithm	Crocker-Grier algorithm	[182]
2007	*Ulva* zoospore	Cell-surface interaction; Swimming motility of *Ulva* zoospores in near-surface and bulk regions	Kirchhoff-Helmholtz transformation	Local extreme sharpness and intensity	Superimposing in-focus reconstructed holograms	[183]
2009	*Ulva* zoospore	Cell-surface interaction; Swimming motility of *Ulva* zoospores in a near-surface region	Kirchhoff-Helmholtz transformation	Local extreme intensity	Satisfying self-consistency between trajectories projected on *xz*- and *yz*-planes	[184]
2012	*Ulva* zoospore	Cell-surface interaction; Swimming motility of *Ulva* zoospores near various surfaces	Kirchhoff-Helmholtz transformation	Local extreme intensity	Satisfying self-consistency between trajectories projected on *xz*- and *yz*-planes	[185]
2006	*Paramecium* species*, Ciliate* species*, Didinium* species*,* and *Coscinodiscus wailesii*	Swimming motility of various microorganisms in ocean or lake environments	Kirchhoff-Helmholtz transformation	Local extreme sharpness and intensity	Superimposing 3D positions of objects and in-focus reconstructed holograms	[26]
2012	*Trypanosoma brucei*	Swimming motility of blood parasites in various external conditions	Kirchhoff-Helmholtz transformation	Local extreme sharpness and intensity	Satisfying self-consistency between trajectories projected on *xz*- and *yz*-planes	[186]
2020	*Haloferax* species and *Haloarcula* species	Comparison of chemotactic behaviors of different *Haloarchaea* species	Rayleigh-Sommerfeld back-propagation	Gouy phase anomaly	Connecting 3D positions for each object in subsequent holograms	[187]
2021	Neuroblastoma	Diving motion of neuroblastoma	Angular spectrum method	Local extreme sharpness	Connecting 3D positions for each object in subsequent holograms	[177]
2021	*Leishmania mexicana*	Comparison of swimming phenotypes of infective and non-infective intracellular parasites	Rayleigh-Sommerfeld back-propagation	Gouy phase anomaly	Connecting 3D positions for each object in subsequent holograms	[188]
2023	MS2 bacteriophage and severe acute respiratory syndrome coronavirus 2	Detection of airborne virus-laden droplets and aerosols	Kirchhoff-Helmholtz transformation	Local extreme sharpness and intensity	Superimposing in-focus reconstructed holograms	[189]

### Label-free identification of biological cells

Holographic images of biological cells can be usefully utilized for label-free cell identification. A lens-free holographic imaging platform was developed for on-chip cytometry to automatically characterize erythrocytes, yeast cells, *E. coli*, and micro-particles of various sizes [190]. A custom-made decision algorithm was introduced to match the detected hologram textures of arbitrary objects with hologram datasets. The lens-free holographic on-chip imaging platform was employed for enumeration and volume measurement of human blood cells, as well as differentiation between various types of white blood cells, including granulocytes, monocytes, and lymphocytes [191]. The three different types of unlabeled leukocytes were classified by evaluating their cellular size and internal complexity [192]. The focal lengths of real focus and virtual focus were determined from light-intensity profiles obtained from reconstructed holographic images of erythrocytes [193]. The real focal length of erythrocytes decreased after the morphological transition from discocytes to echinocytes and spherocytes with the lapse of duration time of blood storage. Breast cancer cells and ovarian cancer cells were enumerated and characterized based on the in-focus scattered light intensity and cell diameter obtained from their holographic images [194]. Bacterial colonies of *E. coli* and *Staphylococcus intermedius* were classified using principal components analysis applied to optical signatures, such as reconstructed amplitude and phase maps [195]. The growth characteristics of *Haematococcus pluvialis* were experimentally analyzed by measuring the variation in cell size under different levels of light stress [196].

With the rapid advancements of AI, a variety of machine learning and deep learning algorithms have been employed for label-free cell classification. A decision tree was employed to classify 3 distinct types of erythrocytes, including discocytes, echinocytes, and spherocytes [197]. To train a machine learning algorithm, numerous features of erythrocytes were quantified, including morphological traits, intensity distributions of holograms, and optical focusing characteristics. The classification of healthy and unstained malaria-infected erythrocytes was achieved by using a support vector machine (SVM) algorithm trained with morphological and light scattering characteristics [198]. Similarly, the SVM algorithm with a linear kernel trained on various features such as basic morphologies, optical characteristics, and translational and rotational invariants was used to classify different types of leukocytes [199].

Label-free classification techniques were developed using a decision tree algorithm trained with characteristic metrics of cell size and intensity values of holograms to enumerate erythrocytes, peripheral blood mononuclear cells, and breast cancer cells [200]. A CNN consisting of 5 convolutional layers was utilized to classify human mammary gland epithelial cells, breast cancer cells, and esophageal cancer cells [201]. In-flow enumeration of breast cancer cells and ovarian cancer cells from lysed blood samples containing white blood cells was performed using a custom-built shallow network [202]. The viability and concentration of yeast cells were evaluated by employing the SVM algorithm trained with spatial features extracted from the reconstructed amplitude and phase maps [203]. A deep-learning-based architecture named You Only Look Once version 5 was employed to directly predict the viability of yeast cells from denoised holograms without a hologram reconstruction process [204]. Diatom phytoplankton, diatom pennate, *Navicula* species, and *Selenastrum* species were classified by a random forest algorithm trained with various features, such as optical volume, coefficient of variation, mean optical path length, projected area, cell skewness, and cell kurtosis [205]. The death rate of algal cells in the East China Sea was assessed using the SVM algorithm trained with features collected from reconstructed amplitude and phase maps obtained from holographic images of *Prorocentrum lima* algae [206]. A 3D CNN model was utilized to measure the number of clustered algae *Phaeodactylum tricornutum* [207]. With the assistance of AI-based DIHM technique, most biological cells can be detected and enumerated with high throughput. These studies on AI-based label-free identification methods for biological cell analysis are summarized in Table 6 [197–207].

**Table 6:** Summary of previous studies on artificial intelligence (AI)-based label-free identification of biological cells using digital in-line holographic microscopy

Year	Object	Content	AI algorithm	References
2018	Erythrocyte	Classification of discocytes, echinocytes, and spherocytes	Decision tree	[197]
2018	Erythrocyte	Classification of healthy and malaria-infected erythrocytes	Support vector machine (SVM)	[198]
2018	Leukocyte	Classification of lymphocytes, granulocytes, and monocytes	SVM with a linear kernel	[199]
2017	Tumor cell	Screening and enumeration of erythrocytes, peripheral blood mononuclear cells, and breast cancer cells	Decision tree	[200]
2021	Tumor cell	Classification of human mammary gland epithelial cells, breast cancer cells, and esophageal cancer cells	Convolutional neural network (CNN)	[201]
2023	Tumor cell	Enumeration of breast cancer cells and ovarian cancer cells	Custom-built shallow network	[202]
2016	Yeast cell	Evaluation of viability and concentration of yeast cells	SVM	[203]
2023	Yeast cell	Evaluation of viability of yeast cells	You Only Look Once version 5	[204]
2018	Diatoms and algae	Automatic identification of various biological cells	Random forest	[205]
2021	*Prorocentrum lima* (*P. lima*)	Evaluation of death rate of algae *P. lima*	SVM	[206]
2022	*Phaeodactylum tricornutum* (*P. tricornutum*)	Enumeration of clustered algae *P. tricornutum*	Three-dimensional CNN	[207]

## Comparison with other 3D imaging techniques

### Comparison with off-axis digital holographic microscopy (DHM)

Off-axis DHM has been extensively investigated for measuring 3D phase information in various biological samples [28]. In the off-axis DHM systems, there exists a slight difference in the propagating directions of reference and object waves. A band-pass filter is commonly employed to separate real, twin, and zero-order images in the frequency domain of a recorded hologram. The off-axis DHM systems have been utilized for quantitative phase imaging [208–210], holographic tomography [211, 212], and dynamic analysis of biological samples [213–216]. On the contrary, obtaining clear real images from holograms recorded by DIHM systems is challenging due to the twin-image problem. To address this issue during the hologram reconstruction process, iterative phase retrieval methods [100, 101, 105] and deep learning techniques [104, 217, 218] have been developed. However, sparse test samples with weak phase fluctuations are prerequisites to ensure high precision in phase retrieval using DIHM systems. Therefore, off-axis DHM is suitable for analyzing detailed 3D morphology of biological samples.

On the other hand, the space bandwidth product (SBP) of reconstructed holograms obtained from an off-axis DHM system is somewhat limited compared to DIHM [219]. The SBP of reconstructed holograms is determined by multiplying the FOV and spatial frequency bandwidth. In comparison, the SBP values for DIHM are approximately three times larger for Fresnel holograms and two times larger for Fourier holograms, compared to those of off-axis DHM. Assuming a fixed FOV in the reconstructed holograms, DIHM has a higher maximum spatial frequency than off-axis DHM. Therefore, the resolvable details of an object in reconstructed holograms from DIHM are finer than those from off-axis DHM. Additionally, high-frequency components in reconstructed holograms from off-axis DHM undergo partial filtration during the extraction process of real images. To determine the depth position of biological samples, an autofocusing function is usually utilized to search for an in-focus image with the highest sharpness or GRA among the reconstructed holograms. Given that the SBP of reconstructed holograms from DIHM is higher than that of off-axis DHM, adopting a DIHM system is advantageous for reconstructing edge structures with high-frequency components and accurately determining their 3D positional information.

The NA of off-axis DHM systems is also limited due to the requirement for a minimum recording distance to distinguish between real, twin, and zero-order images. To overcome this limitation in spatial resolution for off-axis DHM, it is common practice to employ an objective lens with a high magnification ratio and NA to acquire detailed 3D phase information of test samples. However, increasing the magnification ratio of the objective lens leads to a decrease in the FOV of off-axis DHM. On the other hand, DIHM systems typically utilize an objective lens with a relatively lower magnification ratio and NA for analyzing 3D locations of microscale particulates from the reconstructed holograms, while maintaining sufficient spatial resolution. Thus, 3D dynamics of biological samples moving in a wide FOV can be tracked more precisely compared to off-axis DHM. Additionally, due to its simpler optical configuration and shorter recording distance requirements, building up experimental setups for DIHM systems is easier. Therefore, the DIHM system is suitable for the 4D tracking of biological cells.

### Advantages of DIHM over other microscopic imaging techniques

Various microscopic imaging techniques have been developed to visualize the 3D structures of biological samples with high lateral and axial resolutions. Fluorescence microscopy techniques, including confocal microscopy [20], two-photon microscopy [220], multi-photon fluorescence microscopy [221], and structured illumination microscopy [222], enable the reconstruction of 3D morphology by stacking fluorescent images at different focal planes or scanning fluorescence point-by-point across the samples. Light-sheet microscopy is utilized to scan a test sample by irradiating a light sheet at different depths and angles [223]. Spatial light interference microscopy is used to measure nanoscale phase information and dynamics of live cells over periods ranging from seconds to days [224]. Differential-interference-contrast microscopy collects a set of 2D images of a thick sample by moving it through the direction of focus to obtain its 3D image [225]. These advanced 3D imaging techniques effectively visualize the 3D structures and long-term variations of biological samples. However, real-time monitoring of rapidly changing cell dynamics is somewhat limited due to the shallow DOF and the long scanning time associated with these 3D imaging techniques.

Compared to other 3D imaging techniques, DIHM enables simultaneous measurement of multiple biological cells located at different depth-wise positions. It can analyze not only unicellular organisms but also multicellular organisms, including marine plankton [27, 49, 50, 226, 227], embryos [228], and stem cells [229, 230]. Additionally, DIHM allows visualization of transparent thin tissue structures, such as human breast carcinoma [231] and human hepatocellular carcinoma tissues [232]. Unlike the volumetric scanning process used in other techniques, DIHM records holographic images of moving cells using a high-speed camera with a high frame rate. This facilitates effective analysis of the 3D dynamics of biological cells with high temporal resolution.

The specialized nature of DIHM in analyzing the 3D dynamics of individual biological cells makes it a highly effective tool for studying multicellular interactions and collective cell migration behaviors. For example, DIHM has been employed to investigate cell-cell interactions between intestinal pathogenic bacteria *E. sakazakii* and the probiotic *Lactobacillus rhamnosus* [171]. Additionally, it has been utilized to monitor the morphology and migration behaviors of cancer cells in large-scale 3D matrix gels [233]. Consequently, DIHM systems can be effectively used for analyzing cell-cell interactions and cell migrations in various microenvironments that are challenging to investigate with other 3D imaging techniques.

### Limitations of DIHM

Nevertheless, DIHM does have certain technical and experimental limitations. The lateral and axial resolutions are diffraction-limited due to the optical configuration of DIHM systems. The FOV is typically limited to hundreds of microns due to the restricted SBP of DIHM. Moreover, the shallow depth of focus prevents full reconstruction of the morphological structure of elongated objects. Overlapped holographic signals from highly concentrated particles reduce the accuracy of their 3D localization measurements. To avoid unexpected optical aberrations, it is crucial for the medium containing a test sample to have a uniform refractive index and for the beam paths within it to be free from impurities that induce unnecessary light scattering. Therefore, meticulous arrangement of the experimental setup and cautious execution of experiments are essential prerequisites for obtaining clear holographic signals from test samples.

## Conclusions and perspectives

In summary, the DIHM technique holds great promise as a 3D imaging method suitable for label-free identification and tracking of various biological cells at the microscale level. It enables quantitative analysis of diverse 3D dynamics exhibited by biological cells, such as motility, migration, cell-surface interaction, and chemotactic behavior. By acquiring statistical information on their 3D location, orientation, and morphology over time with the aid of DIHM, it is possible to gain insights into these dynamic behaviors. Recent advancements in hologram handling techniques have significantly improved measurement accuracy in characterizing 3D dynamic behaviors of different species of cells under varying conditions such as diseases, external stimuli, and surrounding environments. Notably, rapid progress in AI technology has greatly enhanced the processing speed during reconstruction and autofocusing stages for precise 3D localization, while improving image quality through super-resolution algorithms and twin-image suppression methods. The reconstructed amplitude and phase maps derived from biological cells provide morphological and optical features that can be used for label-free detection, classification, and enumeration.

On the contrary, the majority of reconstruction and autofocusing methods that utilize AI techniques exhibit diminished generalization performance due to the limited availability of training datasets. Although AI techniques can effectively reduce the computational time required for reconstruction and autofocusing procedures, only a few studies have applied them to 3D PTV analysis of biological cells. Given the importance of demonstrating distinct differences in experimental samples and control groups in biological research, it is crucial to rapidly process numerous holographic images to obtain reliable statistical results. Conventional numerical reconstruction equations have been used for decades, as they are capable of reconstructing the amplitude and phase maps at different depth-wise locations from holographic signals of any unknown particles. If an innovative AI model with superior generalization performance were developed to replace conventional reconstruction methods, it would greatly accelerate the time-consuming digital image processing routines for cell tracking and facilitate related biological research.

The lateral and axial resolutions of DIHM are primarily determined by the NA of the DIHM setup and the wavelength of the light source, resulting in minor differences in resolutions among different DIHM systems. These slight variations depend on their specific optical configurations and numerical reconstruction methods. Rayleigh-Sommerfeld back-propagation and angular spectrum method would offer slightly more accurate holograms reconstructed from an original hologram recorded by DIHM systems using plane waves, as they do not require any approximation. Additionally, other numerical reconstruction equations using approximations can be utilized to reduce computational costs.

The main source of measurement errors in the 3D localization of biological cells is primarily associated with the autofocusing process. Although several autofocusing methods have demonstrated excellent performance for their own optical setup in DHM, it should be noted that the optimal focus function may depend on various factors, such as sample type, optical configuration, and post-image-processing method involving denoising and reconstruction algorithms. Therefore, selecting the most suitable autofocusing method for a specific experimental setup often requires trial and error. With the exception of highly concentrated particles, all PTV algorithms generally provide sufficient accuracy in the tracking process. It is recommended to refer to other studies that successfully analyzed similar biological cells to identify an optimal experimental setup and adapt it accordingly to suit specific experimental conditions. Several commercial DIHM platforms, including 4-Deep in-line holographic microscopes (NanoAndMore, USA), HO-DIHM-HT01 (Holmarc Opto-Mechatronics, India), and LISST-Holo2 (Sequoia Scientific, USA), are available for various applications.

By integrating the compact optical configurations of DIHM with state-of-the-art AI algorithms, it is possible to develop user-friendly devices for real-time and in situ analysis of biological cells. Portable smartphones or tablet computers equipped with DIHM and AI techniques can be effectively utilized for the facile diagnosis of hematologic diseases characterized by morphological disorders in human blood cells, as well as for continuous monitoring of hazardous microscale particulates, such as toxic contaminants, bacteria, and viruses in airborne or submersible environments. The ongoing advancements in DIHM and AI techniques hold great potential to significantly enhance the measurement performance related to translational and rotational behaviors, cell deformations, as well as cell-cell and cell-surface interactions. While previous studies on hemodynamics have primarily focused on the translational motions of erythrocytes, innovative AI-based DIHM techniques can now be employed to measure the dynamic behaviors of abnormal erythrocytes, thereby shedding light on their unknown impacts on human health. Furthermore, these advances enable simultaneous measurements of rapid variations in motility, orientation, and morphology of biological cells under various chemical, optical, and mechanical stimuli. Such progress offers high measurement accuracy along with significant throughput capacity and fast processing speed for quantitative analyses of biological cells. By fostering collaboration between engineers utilizing DIHM technologies and cell biologists providing expertise in cellular behaviors, a wide range of new research can be conducted to elucidate previously undisclosed dynamic characteristics of various biological cells, and will provide valuable information for potential applications in the diagnosis and treatment of associated diseases.

## Data Availability

Not applicable.

## Abbreviations

2D: Two-dimensional
3D: Three-dimensional
4D: Four-dimensional
AI: Artificial intelligence
*A. punctulata*: *Arbacia punctulata*
*A. tumefaciens*: *Agrobacterium tumefaciens*
CNN: Convolutional neural network
*C. polykrikoides*: *Cochlodinium polykrikoides*
DHM: Digital holographic microscopy
DIHM: Digital in-line holographic microscopy
DOF: Depth of field
*D. primolecta*: *Dunaliella primolecta*
*E. coli*: *Escherichia coli*
*E. sakazakii*: *Enterobacter sakazakii*
FFT: Fast Fourier transform
FOV: Field of view
GRA: Gradient
*K. veneficum*: *Karlodinium veneficum*
LAP: Laplacian
NA: Numerical aperture
PDF: Probability density function
PTV: Particle tracking velocimetry
*P. aeruginosa*: *Pseudomonas aeruginosa*
*P. lima*: *Prorocentrum lima*
*P. minimum*: *Prorocentrum minimum*
*P. piscicida*: *Pfiesteria piscicida*
*P. tricornutum*: *Phaeodactylum tricornutum*
*P*-value: Probability value
SBP: Space bandwidth product
SPEC: Weighted spectral
SVM: Support vector machine
*S. putrefaciens*: *Shewanella putrefaciens*
TC: Tamura coefficient
TTLL: Tubulin-tyrosine ligase-like
*Ttll*3^−/−^*Ttll*8^−/−^ mouse: A double-knockout mouse for two initiating glycylases TTLL3 and TTLL8
VAR: Variance
